# Application of unconventional microorganisms for the production of non-alcoholic beer

**DOI:** 10.3389/fmicb.2026.1830878

**Published:** 2026-05-22

**Authors:** Selin Yabacı Karaoğlan, Pavel Dostálek

**Affiliations:** 1 Department of Food Engineering, Faculty of Engineering, The University of Adana Alparslan Türkeş Science and Technology, Adana, Türkiye; 2 Department of Biotechnology, Faculty of Food and Biochemical Technology, University of Chemistry and Technology, Prague, Czechia

**Keywords:** aroma, fermentation, brewing, flavor, lactic acid bacteria, maltose-negative, mixed fermentation, non-alcoholic beer

## Abstract

Non-alcoholic beer (NAB) is one of the rapidly expanding segments of the beer market, yet many products still suffer from wort-like sweetness, thin body, and muted hop–yeast complexity. These quality gaps arise because conventional approaches, such as limited fermentation with *Saccharomyces* or physical dealcoholization of regular beer, are fundamentally constrained by the central role of maltose and maltotriose fermentation in standard brewing. Unconventional microorganisms offer an alternative biological route to the production of NAB. Non-*Saccharomyces* and maltose-negative yeasts, lactic acid bacteria, mixed cultures and engineered strains combine restricted utilization of wort sugars with diverse metabolic outputs, including fruity and spicy aroma compounds, organic acids, glycerol and exopolysaccharides that can modulate flavor balance, mouthfeel and pH in the near absence of ethanol. Drawing on recently published pilot-scale and industrial examples, this review synthesizes current knowledge on how these organisms reshape wort sugar metabolism and the resulting volatile and non-volatile profiles in NAB. Particular attention is paid to realistic process concepts such as primary fermentation with maltose-negative yeasts, LAB-assisted fermentations, and restricted-ethanol fermentations using selected or engineered *Saccharomyces* variants. Remaining challenges include robustness in hopped wort, control of off-flavors (diacetyl, ethyl acetate, phenolic notes), regulatory acceptance of engineered strains and consistent sensory quality at scale. Overall, unconventional microorganisms emerge as key tools to close the sensory gap between NAB and regular beer while supporting more diverse, lower-impact brewing practices.

## 1 Introduction

Globally, conventional beer production primarily relies on yeasts belonging to the genus *Saccharomyces* for alcoholic fermentation (Capece et al., 2018). In ale-type beer production, the top-fermenting yeast *Saccharomyces cerevisiae* is predominantly used, whereas lager-type beer (accounting for approximately 90% of global beer production) is produced using *Saccharomyces pastorianus*, a natural hybrid of *S. cerevisiae* and *S. eubayanus*. Unconventional microorganisms encompass all microbial species outside these traditionally used, metabolically well-characterized, and industrially standardized strains and can be sourced from isolated natural sources, hybridization, and genetic modification (Maust et al., 2025). In recent years, several of these microorganisms have been extensively investigated for producing non-alcoholic and low-alcohol beers. Examples include *Saccharomycodes ludwigii* (Maust et al., 2025), *Torulaspora delbrueckii* (Bellut et al., 2018; Nikulin et al., 2022; Canonico et al., 2024), *Cyberlindnera subsufficiens* (Bellut et al., 2019; Nyhan et al., 2023), *Pichia kluyveri* (Vaštík et al., 2022; Maust et al., 2025), species of the genus *Hanseniaspora* (Bellut et al., 2018; Maust et al., 2025), *Lachancea fermentati* (Nyhan et al., 2023), *Starmerella bombicola* (Vaštík et al., 2022), *Kazachstania servazzii*, and *Kluyveromyces marxianus* (Johansson et al., 2021). In addition, lactic acid bacteria such as *Lactiplantibacillus plantarum* (Nyhan et al., 2023) and kefir-derived microbial consortia (do Nascimento et al., 2025) have also been explored as alternative fermentation agents.

The rise of unconventional microorganisms in brewing initially resulted from consumers’ growing interest in more diverse and distinctive aroma profiles (Maust et al., 2025). This trend has been further reinforced over the past decade by the marked global increase in demand for non-alcoholic beer, driven by heightened health awareness, shifting consumption patterns, and increasingly strict regulations on alcohol consumption. Although technological approaches such as dealcoholization and thermal evaporation have traditionally been used to reduce ethanol content, these methods often result in significant loss of aroma compounds and overall sensory complexity, ultimately yielding products that lack the characteristic profile of conventional beers. It should be noted, however, that certain dealcoholization techniques, such as vacuum distillation, membrane contactors and pervaporation, allow for partial aroma recovery. Nevertheless, these methods generally require substantial capital investment, which limits their accessibility for many breweries (Horácio et al., 2020). Consequently, biological strategies aimed at directly limiting ethanol formation during fermentation, particularly through the application of unconventional microorganisms, have attracted increasing scientific and industrial interest (Methner et al., 2022; Simões et al., 2023; Maust et al., 2025).

The definition of NAB and its legal thresholds vary across countries and regulatory frameworks. In many jurisdictions, the upper limit for products labeled as “non-alcoholic” is set at ≤0.5% alcohol by volume (ABV), whereas stricter thresholds (e.g., ≤0.05% or ≤0.1% ABV) apply in certain regulations for the use of the term “alcohol-free” (e.g., the United States and the United Kingdom) (De Francesco et al., 2015; Maust et al., 2025; Schubert and Rettberg, 2025). These terminological differences arise from both national legislation and marketing strategies, and they may complicate the classification and comparison of scientific results. Indeed, some experimental studies categorize beers containing ≤0.5% (v/v) ethanol as “non-alcoholic” (Methner et al., 2022; Rettberg et al., 2022; Simon et al., 2024), whereas similar alcohol levels are classified as “low-alcohol” in other studies (Vaštík et al., 2023). This inconsistency makes it critical to clearly define the targeted alcohol level when evaluating the performance of biologically produced products. In experimental brewing research, ≤0.5% (v/v) ABV is most commonly used as the operational definition of “non-alcoholic” beer (Bellut et al., 2019; Methner et al., 2022). For “low-alcohol” beer, an ABV range of 0.5–1.2% has been reported as a commonly applied framework in most European Union countries (Simon et al., 2024). For clarity, the present review adopts the ≤0.5% (v/v) ABV threshold when referring to non-alcoholic beer, unless otherwise specified.

NAB production can be broadly classified into two main approaches: (i) physical dealcoholization methods involving post-fermentation ethanol removal and (ii) biological strategies aimed at limiting ethanol formation during fermentation (De Francesco et al., 2015; Simões et al., 2023; Maust et al., 2025; Schubert and Rettberg, 2025). Although physical methods, such as membrane-based systems, vacuum distillation/rectification, and reverse osmosis, are effective in reducing ethanol content, they are known to cause sensory drawbacks, particularly at low- and non-alcoholic targets, due to the concomitant loss of volatile aroma compounds (Brányik et al., 2012; De Francesco et al., 2015; Rettberg et al., 2022; Schubert and Rettberg, 2025). In contrast, biological approaches aim to limit ethanol formation during fermentation by controlling wort fermentability or by modulating microbial metabolism while maintaining desirable sensory characteristics. These strategies include process-based interventions such as modification of the mashing regime, cold contact fermentation, and limited fermentation, as well as microbiological approaches involving the use of unconventional microorganisms (Nyhan et al., 2023). Among biological approaches, the use of unconventional microorganisms has emerged as a promising alternative for NAB production (Simon et al., 2024; Maust et al., 2025).

Among the various biological approaches, particular attention has been paid to strategies that directly involve microbial application. A review of the literature indicates that these fermentation-based studies can be broadly categorized according to their ethanol-reduction strategies. In these studies, ethanol reduction is primarily achieved by exploiting restricted carbohydrate metabolism, altered respiratory behavior, or controlled fermentation kinetics. Accordingly, the reported approaches include: (i) screening and application of non-*Saccharomyces* yeasts (Methner et al., 2022; Vaštík et al., 2022; Klimczak et al., 2024); (ii) the use of maltose-negative *Saccharomyces* strains, including naturally occurring and engineered variants (Nyhan et al., 2023; Vaštík et al., 2023; Simon et al., 2024; Maust et al., 2025); (iii) co-fermentation strategies involving lactic acid bacteria (LAB) (Nyhan et al., 2023) or mixed microbial consortia (do Nascimento et al., 2025); and functionally oriented inoculations, including probiotic-targeted fermentations (Vaštík et al., 2025). In the literature, it is evident that quality in non-alcoholic beers is constrained along three principal chemical and technological axes: (i) aldehydes and the worty off-flavor, (ii) sweetness and body arising from residual sugars, and (iii) clarity, filtration, and storage stability (Bellut et al., 2019; Rettberg et al., 2022; Schubert and Rettberg, 2025). Several studies have directly addressed the observation that, in non-alcoholic beers produced through limited fermentation, low ethanol content combined with elevated residual sugars can intensify the perception of a ‘wort-like’ character, a defect closely associated with aldehydes that may be masked through ester production or mitigated by enhancing aldehyde-reducing capacity (Bellut et al., 2018; Nikulin et al., 2022; Nyhan et al., 2023; Maust et al., 2025).

In this context, biological approaches that move beyond conventional *Saccharomyces* fermentation have attracted growing attention in NAB production in recent years. Unconventional microorganisms in particular, offer distinct aroma metabolisms coupled with limited sugar utilization, and thus hold considerable potential for achieving a more balanced sensory profile even under low-ethanol conditions. However, the existing body of research has largely been conducted under varying experimental conditions, with substantial differences in wort composition, fermentation strategies, and analytical assessment methods. This heterogeneity makes direct comparison of findings across the literature considerably difficult. The aim of this review is therefore to critically evaluate the current literature on the use of unconventional microorganisms in NAB production within a systematic framework. The review specifically addresses biological strategies developed around non-*Saccharomyces* yeasts, maltose-negative *Saccharomyces* strains including naturally occurring and engineered variants, lactic acid bacteria, mixed microbial consortia, and probiotic-targeted fermentation approaches, discussing the effects of these microorganisms on sugar metabolism, aroma formation, mouthfeel, stability, and microbiological safety.

## 2 Application strategies experimentally tested in NAB production

### 2.1 Wort composition and fermentability control

Wort composition is a critical variable in NAB production, directly determining both fermentation performance and the sensory profile of the final product (Brányik et al., 2012; Piornos et al., 2023). Within the carbohydrate profile of wort, maltose is the predominant fermentable sugar, accounting for approximately 70% of the total, and this high maltose content represents one of the principal factors complicating the limitation of alcohol formation in NAB production (Bellut and Arendt, 2019; Basu et al., 2026). In NAB production, the original gravity (OG) of wort is generally targeted at 5–7 °P. This low-gravity approach restricts the amount of fermentable sugar and, consequently, the potential ethanol yield from the outset (Basu et al., 2026). It should be noted, however, that higher OG values have also been applied in maltose-negative yeast strategies (Basu et al., 2026). Raising the mash temperature (≥72 °C, isothermal) substantially reduces *β*-amylase activity while allowing *α*-amylase to predominate, thereby increasing the production of unfermentable dextrins and reducing ethanol content to as low as 2.8% v/v compared to conventional processing (Endres et al., 2022; Fritzsche et al., 2025; Basu et al., 2026). Alternatively, cold mashing (~10 °C) restricts enzymatic starch hydrolysis, lowering the fermentable sugar concentration of the wort to approximately 5 °Brix (~5 °P) and providing a suitable starting point for low-ethanol production (Dalberto et al., 2023). It is also noteworthy that the low processing temperature suppresses Maillard reactions and Strecker degradation, making this technique particularly relevant as a strategy capable of simultaneously reducing both fermentable sugar content and, to some extent, aldehyde load (Basu et al., 2026).

Free amino nitrogen (FAN) is a key determinant of yeast metabolism and aroma formation, influencing the synthesis of higher alcohols, esters, and other fermentation-derived volatiles (Hill and Stewart, 2019). Insufficient FAN (below approximately 130 mg/L) has been associated with sulfide evolution, incomplete fermentation, and reduced ester production, whereas excess FAN is linked to the accumulation of off-flavors such as diacetyl and fusel alcohols. The optimal range for standard gravity fermentations is generally cited as 200–250 mg/L (Hill and Stewart, 2019). It has further been reported that in low-gravity worts (≤7 °P), FAN levels can decline to 55–99 mg/L. However, due to reduced fermentation activity, the yeast’s FAN requirement also decreases proportionally. Accordingly, optimizing the balance between wort original gravity and FAN concentration is recommended (Basu et al., 2026).

From a mouthfeel perspective, ethanol is known to contribute to this perception through three distinct mechanisms: (i) increasing liquid viscosity and thereby creating a physical sense of fullness, (ii) generating a characteristic warmth and burning sensation in the mouth, and (iii) acting as an aroma carrier by facilitating the transport of volatile compounds to the retronasal passage (Piornos et al., 2023; Wolinska-Kennard et al., 2025). In NAB production, all three of these effects are negligible or absent; furthermore, glycerol, a yeast fermentation by-product that contributes to mouthfeel fullness and smoothness, is generally present at low concentrations [typically 0.4–1.0 g/L in biologically produced NABs, compared to 1–2 g/L in conventional beer (Vaštík et al., 2022; Vaštík et al., 2023)], primarily due to the reduced original gravity and limited wort fermentability associated with biological production methods of NABs (Wolinska-Kennard et al., 2025; Basu et al., 2026; Myncke et al., 2026).

To compensate for these losses, the simultaneous enhancement of dextrin and high molecular weight arabinoxylan (HMW-AX) in wort has been reported as an effective strategy, with the individual increase of either compound alone shown to be insufficient to achieve meaningful sensory improvement (Michiels et al., 2025). The combination of dextrin and HMW-AX appears to partially address the first mechanism (viscosity/physical fullness), with glycerol similarly contributing to this dimension (Basu et al., 2026). However, the second mechanism, the warming sensation in the mouth, cannot be fully substituted in NAB production, and remains one of the fundamental sensory research gaps in the field. Although the supporting literature remains limited, the addition of lactose (milk sugar) has been reported to be occasionally employed in brewing practice with the aim of enhancing mouthfeel fullness and imparting a creamy texture to the final product (Salanță et al., 2020). As lactose is not fermented by yeast and therefore persists as a residual sugar in the final beer, it is regarded as a contributor to perceived body and sweetness and has been notably employed in certain craft beer styles such as “milk stout,” “New England IPA”, and “milkshake IPA.” Nevertheless, systematic academic evidence demonstrating the efficacy of this approach specifically within the context of NAB production remains scarce, and brewers should also consider that lactose is a recognized allergen requiring appropriate consumer labeling (Salanță et al., 2020).

Among the principal sources of off-flavors in NABs, Strecker aldehydes (including 3-methylbutanal, methional, and phenylacetaldehyde) have been reported to be partially reduced at the wort design stage through the application of cold mashing, which suppresses both the Maillard reaction and Strecker degradation (Gernat et al., 2020; Dalberto et al., 2023). Another wort composition parameter that influences aroma balance is hop dosage. In conventional beers, ethanol partially suppresses the perception of bitterness. However, as this suppressive effect is absent in non-alcoholic beers, an equivalent quantity of hops tends to produce a more pronounced bitterness sensation. For this reason, it is generally recommended that hop dosage be kept lower in NAB production relative to that applied in traditional beer styles (Gernat et al., 2020; Piornos et al., 2023). Table 1 presents the wort parameters commonly recommended for NAB production. In addition to wort composition, the final pH of non-alcoholic beers typically remains within the range of approximately 4.3–4.6, which contributes to microbiological stability and overall sensory balance (Myncke et al., 2026). Overall, wort design represents the first technological barrier to ethanol formation in NAB production. However, controlling wort composition alone is rarely sufficient to achieve both low ethanol levels and acceptable sensory quality. Consequently, microbial strategies based on unconventional microorganisms have gained increasing attention.

**Table 1:** Target wort parameters recommended for optimal NAB production.

Parameter	Recommended range in literature for NAB (<0.5% ABV)	Purpose	References
Original Gravity (OG)	5–7 °P	To limit ethanol formation. Note: Applicable to low-gravity mash strategies; higher OG values may be employed when maltose-negative yeast strategies are applied.	Basu et al. (2026) and Myncke et al. (2026)
Mashing Temperature	Hot mashing: 75–80 °C (isothermal) Cold mashing: ~10 °C	To increase the dextrin content by denaturing *β*-amylase To simultaneously reduce the load of fermentable sugars and aldehydes by restricting enzymatic hydrolysis	Endres et al. (2022) and Dalberto et al. (2023)
Dextrin	>30 g/L	To enhance the perception of body in the mouth	Michiels et al. (2025)
HMW-AX	>1.5 g/L	To support viscosity and body perception	Michiels et al. (2025)
Free Amino Nitrogen (FAN)	~200–250 mg/L for conventional beers / For NAB fermentation at approximately 7 °P: 55–99 mg/L (low but proportionally sufficient)	To support yeast metabolism and aroma formation	Basu et al. (2026)
pH	5.2–5.5	To optimize enzyme activity	Methner et al. (2022) and Myncke et al. (2026)
Hop Bitterness (IBU)	~10–25	To balance sweetness and flavor	Basu et al. (2026) and Myncke et al. (2026)

NAB, non-alcoholic beer (<0.5% ABV); HMW-AX, high molecular weight arabinoxylan; IBU, International Bitterness Units.

### 2.2 Microbial strategies

In the literature, microbial strategies for NAB production can broadly be grouped under two practical approaches. The first encompasses yeast-based strategies founded on the restriction of carbohydrate utilization. This approach relies principally on the use of yeasts that are unable to ferment, or can only limitedly utilize, maltose, the primary sugar in wort, thereby naturally constraining the conversion of sugars to ethanol and enabling the production of beers with very low alcohol content. Within this framework, non-*Saccharomyces* species with maltose-negative or limited maltose-utilizing capacity, as well as maltose-negative *Saccharomyces* strains and their hybrids, have been frequently investigated in the literature (Simões et al., 2023; Maust et al., 2025). The second group comprises co-fermentation or biological acidification-based approaches involving lactic acid bacteria (LAB) and probiotic bacteria. In these systems, LAB metabolism can indirectly constrain yeast fermentation activity by lowering the pH of the fermentation environment, while simultaneously influencing the sensory characteristics of the final product. In certain studies, this approach has also been evaluated for its potential to impart functional or probiotic properties to the finished beer (Nyhan et al., 2023; do Nascimento et al., 2025; Vaštík et al., 2025).

#### 2.2.1 Yeast-based strategies relying on restricted carbohydrate utilization

##### 2.2.1.1 Application of non-*Saccharomyces* yeasts

The majority of studies focusing on the use of non-*Saccharomyces* yeasts in NAB production exploit the characteristic inability or limited capacity of these species to utilize maltose and maltotriose, the principal fermentable sugars present in brewing wort. This property naturally constrains fermentation, enabling the production of beers with low alcohol content (Bellut et al., 2018; Vaštík et al., 2022; Maust et al., 2025). The diversity of species and strains investigated within this strategy is considerable. The literature reports the use of yeasts isolated from distinct ecological niches, including kombucha (Bellut et al., 2018) and sourdough (Johansson et al., 2021), alongside strains obtained from non-*Saccharomyces* screening collections (Vaštík et al., 2022), as well as commercial yeast preparations specifically developed for NAB production (Methner et al., 2022; Maust et al., 2025). Supplementary Table S1 provides a consolidated overview of the non-*Saccharomyces* yeast strategies reported in the literature, together with the key characteristics of the resulting products.

Among the non-*Saccharomyces* yeasts evaluated for NAB production, a consistent technological advantage is their limited ability to ferment the dominant wort sugars maltose and/or maltotriose, which allows ethanol formation to remain within or close to the NAB range under appropriately designed wort and process conditions. However, the available studies also show that this metabolic restriction does not lead to a uniform sensory outcome. Instead, the resulting beers differ markedly in aroma formation, residual sweetness, aldehyde reduction capacity, body, and process robustness, indicating that non-*Saccharomyces* yeasts should not be treated as a single functional group but rather as a metabolically diverse set of brewing tools. A recurring pattern in the literature is that ester-driven strains can partially compensate for the low-ethanol environment by generating fruity or floral top notes that reduce the sensory dominance of wort-like character. This is particularly evident for *Pichia kluyveri* and *Hanseniaspora* spp. In the comparative pilot-scale study of Myncke et al. (2025), the commercial *P. kluyveri* strains SMARTBEV™ NEER®, Poly and Punch produced some of the lowest alcohol levels among the tested commercial yeasts (approximately 0.12–0.26% v/v), while simultaneously generating the highest acetate ester concentrations, resulting in beers described as fruity and sweetish rather than neutral or strongly worty. These strains also assimilated little FAN and produced relatively low levels of higher alcohols, indicating a restricted but aroma-relevant metabolism. However, this behavior cannot be generalized uncritically. In Maust et al. (2025), a commercial *P. kluyveri* strain (NEER Punch, Novonesis) was instead described as a low-aroma sample with only slight grape-like character, despite remaining NAB-compliant at both wort strengths. The authors explicitly linked this limited aroma expression to the absence of manufacturer-recommended aeration/agitation during fermentation. Taken together, these findings indicate that the flavor expression of *P. kluyveri* in NAB is not only strain-dependent but also highly process-dependent, particularly with respect to oxygen availability and agitation regime. Based on current brewing literature, a dissolved oxygen concentration of 7–12 mg/L before pitching is generally recommended for conventional fermentations (Basu et al., 2026). Whether similar or higher levels are required for non-*Saccharomyces* yeast fermentations in NAB production remains to be systematically investigated, as oxygen availability has been shown to significantly affect carbon flux and aroma compound formation in Crabtree-negative yeasts (Basu et al., 2026).

Taken together, the literature suggests that non-*Saccharomyces* strategies in NAB production tend to follow two broad functional outcomes. In some cases, strains generate sufficient ester-derived fruity or floral aromas to partially compensate for the sensory role of ethanol, thereby reducing the perception of wort-like character. In other cases, ethanol formation is successfully limited but without adequate sensory compensation, resulting in beers that remain neutral or dominated by wort-like or cereal notes. From an industrial perspective, the former outcome appears more promising; however, even ester-producing strains often remain highly process-dependent. Differences in wort gravity, pH adjustment, oxygen availability, agitation regime, maturation conditions, and post-fermentation stabilization can all influence the final balance between fruity aroma expression, residual sweetness, mouthfeel, and off-flavor formation. Moreover, direct comparison of results across studies remains difficult because experiments have been conducted under widely differing wort compositions, fermentation scales, and sensory evaluation approaches, ranging from trained descriptive panels to untrained industrial RATA (Rate-All-That-Apply) assessments. Consequently, strain selection for NAB production should not rely solely on ethanol yield but rather on the integrated evaluation of volatile profile, aldehyde reduction capacity, mouthfeel contribution, and process robustness under brewery-relevant conditions (Bellut and Arendt, 2019; Rettberg et al., 2022).

Several additional yeast species have been reported in the literature as potential candidates for low-alcohol beer production but were not included in Supplementary Table S1 due to limitations related to alcohol yield, sensory quality, or experimental design. For example, maltose-negative strains isolated from sourdough cultures such as *Kazachstania servazzii* and *Pichia fermentans* have been evaluated at 10 L scale. *P. fermentans* produced a characteristic spicy/clove aroma associated with 4-vinylguaiacol formation, suggesting potential suitability for wheat-style low-alcohol beers, while *K. servazzii* showed good low-temperature tolerance and a relatively clean flavor profile, indicating possible applicability in lager-like products (Johansson et al., 2021). However, the final alcohol levels reported in that study (0.73, 0.52, and 0.68% ABV for *K. servazzii*, *P. fermentans*, and *S. ludwigii*, respectively) exceeded the legal alcohol-free beer threshold of ≤0.5%. Similarly, *Lachancea kluyveri* 3,082 and *Torulaspora delbrueckii* 1I6 tested by Methner et al. (2022) produced 0.55 and 0.54% ABV, respectively. The authors suggested that process adjustments such as lowering original wort extract, shortening fermentation time, or reducing fermentation temperature could potentially bring ethanol production below the regulatory limit. In other cases, strains fulfilling the alcohol criterion were excluded due to quality limitations. For instance, the engineered *Saccharomyces cerevisiae* ΔCIT1 strain produced ≤0.5% ABV but generated acetic acid concentrations exceeding the sensory threshold (200 mg/L), resulting in an undesirable flavor profile (Vaštík et al., 2023). Additional findings highlight practical process constraints associated with some candidate yeasts. Simon et al. (2024) demonstrated that *Saccharomyces cerevisiae* var. *chevalieri* SafBrew™ LA-01 showed a high release efficiency of polyfunctional thiols from glutathionylated hop precursors; however, this observation was based on spiking experiments and still requires confirmation under realistic brewing conditions. Furthermore, Myncke et al. (2026) reported contamination by *Pectinatus* and/or *Megasphaera* in all fermentations performed with two commercial *S. ludwigii* strains (WSL-17 and WLP618) at 20 L scale under low-alcohol conditions, which prevented sensory evaluation and illustrates the microbiological vulnerability of such fermentations due to high residual sugar levels, low ethanol content, and relatively elevated pH. It should be noted, however, that microbiological stability in NABs is highly dependent on post-fermentation treatments such as pasteurization, as well as storage conditions. The contamination events reported in these studies may reflect suboptimal processing or storage rather than an inherent instability of the product. Under appropriate production and storage conditions, non-alcoholic beers can be considered microbiologically stable (Britton and Hill, 2025).

##### 2.2.1.2 Maltose-negative *Saccharomyces* strains and interspecific hybrids

In the literature, the maltose/maltotriose-negative *Saccharomyces* approach has been investigated in parallel with non-*Saccharomyces* strategies, with the shared objective of limiting ethanol formation while diversifying fermentative aroma profiles (Simon et al., 2024; Maust et al., 2025; Myncke et al., 2025). This strategy also represents a distinct application pathway for integrating commercial low- and non-alcoholic *Saccharomyces* strains into brewing processes and for evaluating their interaction with industrial processing steps such as filtration and stabilization.

In contrast to the broader non-*Saccharomyces* group, maltose-negative *Saccharomyces* strains and interspecific hybrids represent a more targeted strategy for NAB production, combining restricted carbohydrate utilization with a closer technological alignment to conventional brewing practice. However, the studies summarized in Table 2 show that this group is functionally heterogeneous. Some strains remain largely neutral and are primarily valuable for ethanol limitation, whereas others provide more specific sensory functions through phenolic aroma formation, polyfunctional thiol release, altered organic acid metabolism, or targeted metabolic engineering.

**Table 2:** Maltose-negative *Saccharomyces* strains and interspecific hybrids strategies experimentally applied for the biological production of non-alcoholic beer.

Microorganism	Mechanism/Key metabolites	Fermentation conditions	Sensory effects	ABV (% v/v)	Notes/Limitations	References
*S. mikatae* CBS 8839 T (wild type)	Maltose/maltotriose-negative; sucrose positive; glucose sole fermentable carbon source; 2-methyl-1-propanol (1.381 mg/L); 2-phenylethanol (0.533 mg/L); ethyl hexanoate (0.274 mg/L — slightly above threshold); 4-vinylguaiacol (0.466 mg/L — above threshold)	7°P hopped wort; 18 °C (1 day) → 3 °C (1 month); 10^6^ cells/mL; 500 mL PET flask; triplicates	No sensory panel conducted; VOC profile neutral; no negative off-flavors reported; 4-vinylguaiacol slightly above threshold (0.2–0.3 mg/L) — typically associated with clove-like/spicy aroma; authors report no flavor impact but no sensory panel conducted to verify	0.03	First reported use of *S. mikatae* in NAB production; wild-type Saccharomyces (non-*cerevisiae*); neutral VOC profile limits aroma contribution; suggested for co-fermentation with flavor-active strains	Vaštík et al. (2023)
Hyb1CIT1 (*S. mikatae* CBS 8839 T *× S. cerevisiae* ΔCIT1)	Maltose/maltotriose-negative; sucrose positive; recombinant mtDNA inherited from both parents; elevated organic acids (citric 0.23 g/L, malic 0.55 g/L); no acetic acid production (unlike parental CIT1); higher alcohols dominant over esters	7°P hopped wort; 18 °C (1 day) → 3 °C (1 month); 10^6^ cells/mL; 500 mL PET flask; triplicates	No sensory panel conducted; VOC profile neutral; no negative off-flavors; slightly higher 3-methyl-1-butanol, 2-methyl-1-propanol and 2-methyl-1-butanol than parental strains but below flavor thresholds; 4-vinylguaiacol slightly above threshold — typically associated with clove-like/spicy aroma; authors report no flavor impact but no sensory panel conducted to verify	0.02	Interspecific hybrid (GMO × wild-type); acetic acid problem of parental CIT1 successfully eliminated; recombinant mtDNA feature; reduced osmotolerance compared to parental strains; no sensory validation; proof-of-concept scale only	Vaštík et al. (2023)
*Saccharomyces cerevisiae* var. *chevalieri* (Fermentis by Lesaffre) SafBrew™ LA-01	Maltose/maltotriose-negative; sucrose partially fermented (81% consumed); glucose and fructose fully consumed; POF + — 4-vinylguaiacol (810 μg/L, ~6.5 × threshold); 4-vinylphenol (642 μg/L, ~3.8 × threshold); total esters 1.5 mg/L and total higher alcohols 32.6 mg/L (all below flavor thresholds); DMS 5 μg/L (below NAB threshold of 48 μg/L)	6°P unhopped wort; 20 °C; 3 days (+3 days 4 °C); 5 × 10^6^ cells/mL; 250 mL; duplicate	Not formally assessed under NAB conditions; POF + character expected to impart white beer/clove-like spicy aroma	0.4%	Commercial strain (Fermentis by Lesaffre); primary fermentation focus of study was polyfunctional thiol release from spiked hop precursors — standard sensory evaluation not conducted; POF + character may not be desirable in all beer styles	Simon et al. (2024)
*Saccharomyces cerevisiae var. chevalieri* (SafBrew™ LA-01, Fermentis)	maltose-negative; POF+	6.37 ± 0.16 °P; mash 72 °C/60 min; 20 °C; 2 days fermentation + 7 days maturation at 3 °C; 4 × 10^6^ CFU/mL (dry yeast, 11 g/20 L); 20 L conical stainless-steel fermenter; wort pH not pre-adjusted (beer pH 4.79 ± 0.16); filtration (2 μm), CO₂ saturation (5.6 g/L), batch pasteurization (50 PU); duplicate fermentations; 6.5 °P target wort	diacetyl 34.7 μg/L (above 17 μg/L threshold); phenolic character driven by 4-VG confirmed sensorially; buttery off-flavor perceived above panel mean.	0.46%	Independent pilot-scale confirmation of Simon et al. (2024) findings; diacetyl exceeds 17 μg/L sensory threshold — extended maturation recommended but limited by contamination risk at low ABV; 4-VG data from preliminary trials only, not quantified in final beers; low glycerol (265 mg/L) contributes to thin body; untrained industrial panel (*n* = 44, Belgian/Dutch); RATA methodology; no contamination observed (unlike co-tested *S. ludwigii* strains)	Myncke et al. (2026)
Berkeley Yeast NA Cabana — genetically engineered *S. cerevisiae*	Maltose-negative; Glucose/fructose fermentation only; tropical fruit character; 3-mercaptohexanol 3-Mercaptohexanol: detected below GC–MS/MS LOD but sensorially active; geranyl acetone and 3-methyl-3-butenol detected at 4.5°P; aldehyde reduction partial; Ethyl caproate: 4.5°P 25.4 μg/L → 9°P 59.5 μg/L (tropical fruit driver)	4.5°P and 9°P wort; 10 BU; 20 °C; 108 h; 1 × 10^7^ cells/mL*; pilot scale (20-gallon); thermal pasteurization (400 PU); triplicate sensory	Pale ale-like; tropical fruit aroma (highest score: 5.48 (4.5°P)); aroma intensity similar at both wort strengths; 9°P increased mouthfeel and overall aroma intensity	4.5°P: 0.36%; 9°P: 0.79% (exceeds NAB limit)	NAB-compliant only at 4.5°P; likely thiol-driven aroma, thiols below LOD but sensorially perceptible; genetic modification not permitted in EU	Maust et al. (2025)
Berkeley Yeast NA Classic — genetically engineered *S. cerevisiae*	Maltose-negative; Glucose/fructose fermentation; engineered terpenoid biosynthesis pathway; citrus/floral character driven by engineered terpenoid production (geraniol, linalyl acetate, citral); terpenoid-based aroma mechanism distinct from ester-driven non-*Saccharomyces* yeasts. Linalyl acetate: 4.5°P 0.241 mg/L → 9°P 0.413 mg/L; Geraniol: ~0.15–0.17 mg/L (in both)	4.5°P and 9°P wort; 10 BU; 20 °C; 108 h; 1 × 10^7^ cells/mL*; pilot scale (20-gallon); thermal pasteurization (400 PU); triplicate sensory	Pale ale-like; citrus and floral aroma; highest floral score: 4.38 (4.5°P) aroma intensity similar at 4.5°P and 9°P; 9°P increased mouthfeel	4.5°P: 0.36%; 9°P: 0.78% (exceeds NAB limit)	NAB-compliant only at 4.5°P; terpene-driven aroma mechanism differs from conventional ester-based flavor formation in non-*Saccharomyces* NAB fermentations; genetic modification not permitted in EU	Maust et al. (2025)
LalBrew® LoNa™ (Lallemand Brewing) — *S. cerevisiae* hybrid	Maltose-negative; Glucose/fructose fermentation; short-chain fatty acids 3-methylbutanoic acid and 2-methylbutanoic acid + ethyl esters (ethyl 3-methylbutyrate, ethyl 2-methylbutyrate) → cheesy aroma; poor flocculation implicated in heat-induced autolysis during pasteurization; 3-Methylbutanal 0.044 mg/L → 3-methylbutanoic acid	4.5°P and 9°P wort; 10 BU; 20 °C; 108 h; 1 × 10^7^ cells/mL*; pilot scale (20-gallon); thermal pasteurization (400 PU); triplicate sensory	Cheesy aroma (highest cheesy score: 5.96 (4.5°P)); low clarity due to poor flocculation; aroma intensity higher at 9°P	4.5°P: 0.37%; 9°P: 0.75% (exceeds NAB limit)	NAB-compliant only at 4.5°P; −cheesy off-note potentially linked to short-chain fatty acid formation and yeast autolysis during pasteurization; filtration prior to pasteurization suggested. Screening of strains with improved FLO1-associated flocculation traits may help mitigate this defect. Because 3-methylbutanoic acid has previously been reported as a fermentation metabolite of *S. cerevisiae*, the extent to which the observed cheesy aroma originates from yeast autolysis versus primary metabolic activity remains uncertain.	Maust et al. (2025)
OYR-252 (Omega Yeast) genetically modified Kveik *S. cerevisiae*	Maltose-negative; Glucose/fructose fermentation; + engineered for high organic acid production; largest pH drop among maltose-negative yeasts tested (Δ1.24 ± 0.050); elevated aldehyde concentrations associated with cereal/wort aroma; sour taste dominated sensory perception; no lactic acid addition required post-fermentation	4.5°P and 9°P wort; 10 BU; 20 °C; 108 h; 1 × 10^7^ cells/mL*; pilot scale (20-gallon); thermal pasteurization (400 PU); triplicate sensory	Strongly sour taste; highest sour taste score: 6.04 (4.5°P), 6.43 (9°P); pH: 4.5°P 3.36, 9°P 3.32; cereal/wort aroma (elevated aldehydes); panelists noted sour beer character; not rated lager-like despite aldehyde profile; high cooked potato (methional) score: 1.28	4.5°P: 0.21%; 9°P: 0.42% — NAB-compliant at both wort strengths	Only GM strain in study compliant at both 9°P and 4.5°P; pH < 4.2 achieved naturally — additional food safety hurdle for NAB; elevated aldehydes contributed to cereal/wort character; strong sourness may restrict appeal outside sour beer style; genetic modification not permitted in EU	Maust et al. (2025)

mtDNA, mitochondrial DNA, the genetic material found in mitochondria, inherited from both parental strains in hybrid Hyb1CIT1 through recombination. DMS, Dimethyl sulfide, a volatile sulfur compound associated with cooked vegetable off-flavors in beer, Kveik, Norvey farmhouse brewer’s yeast, IBU, International Bitterness Units, EBU, European Bitterness Units. *Pitch rates originally reported as “10 million cells/mL” in the cited studies have been expressed as 1 × 10^7^ cells/mL for consistency with the notation used throughout this manuscript.

A representative commercial example is *Saccharomyces cerevisiae* var. *chevalieri* SafBrew™ LA-01. Under non-alcoholic and low-alcoholic beer (NABLAB) fermentation conditions, this strain consumed glucose, fructose, and most of the sucrose while leaving maltose and maltotriose unfermented, producing approximately 0.4% (v/v) ethanol together with low total ester levels and a pronounced POF + phenotype characterized by elevated 4-vinylguaiacol (Simon et al., 2024). Importantly, the same study also showed that LA-01 released polyfunctional thiols from glutathionylated hop precursors with unexpectedly high efficiency, suggesting a potential route for aroma bio-enrichment beyond conventional ester formation, although this observation was obtained under precursor-spiking conditions and still requires confirmation in realistic brewing systems. In a separate pilot-scale comparison, LA-01 again produced NAB-level alcohol (~0.46% v/v), but the resulting beer was characterized more by phenolic notes and elevated diacetyl than by fruity fermentation aroma, illustrating that ethanol compliance alone does not guarantee optimal sensory balance (Myncke et al., 2025).

A second subgroup comprises hybrid and genetically engineered *Saccharomyces* strains designed to introduce more specific metabolic functions. In the work of Vaštík et al. (2023), interspecific hybrids involving *S. mikatae* and maltose-negative *Saccharomyces* backgrounds achieved very low ethanol formation while improving the organic acid profile without increasing acetic acid, yielding relatively neutral volatile profiles rather than strongly aroma-driven beers. By contrast, the pilot-scale study of Maust et al. (2025) demonstrated that engineered maltose-negative *S. cerevisiae* derivatives can be used more explicitly as flavor-design tools: NA Cabana favored tropical fruit expression, likely through thiol-associated aroma pathways; NA Classic generated citrus and floral notes through engineered terpenoid biosynthesis; and OYR-252 combined alcohol limitation with enhanced organic acid production, resulting in a sour NAB with naturally reduced pH. These examples illustrate how maltose-negative *Saccharomyces* strains are increasingly being developed as platforms for targeted metabolic design rather than merely as ethanol-limiting yeasts. As metabolic engineering tools continue to expand, the diversity and performance of such strains are likely to increase further. However, despite their technological potential, the industrial application of genetically modified brewing yeasts remains restricted in several regions, particularly within the European Union, where their use in commercial beer production is currently not permitted.

#### 2.2.2 Lactic acid bacteria and probiotic-oriented fermentation strategies (LAB-assisted fermentations, co-fermentation approaches, acidification and masking of wort-like off-flavors)

Compared with yeast-only ethanol-limiting strategies, LAB/probiotic-oriented approaches in NAB production address a broader technological objective by simultaneously influencing acidification, aroma balance, and, in some cases, functional value. The studies summarized in Table 3 indicate that LAB/probiotic-oriented strategies currently appear in two distinct forms: first, co-fermentation with non-*Saccharomyces* yeasts to modify pH and sensory balance during primary fermentation; and second, post-fermentative enrichment of finished NAB with complex probiotic consortia, such as kefir grains, to enhance microbial functionality while maintaining sensory acceptance (Nyhan et al., 2023; do Nascimento et al., 2025).

**Table 3:** Lactic acid bacteria and probiotic-oriented fermentation strategies experimentally applied for the biological production of non-alcoholic beer.

Microorganism	Mechanism/Key metabolites	Fermentation conditions	Sensory effects	ABV (% v/v), pH, TTA*	Notes / Limitations	Reference
*Lachancea fermentati KBI 12.1* + *Lactiplantibacillus plantarum* FST 1.7	Co-fermentation Limited fermentation (maltotriose-negative yeast) + LAB acidification Key metabolites: lactic acid 1.13 g/L; 2-phenylethanol 5.21 mg/L; diacetyl 0.03 mg/L (below 0.1 mg/L threshold) LAB citrate metabolism → acetoin 6.19 mg/L (↑ vs. yeast alone)	25 °C, 24 h; 6°P unhopped Yeast: 1 × 10^6^ CFU/mL; LAB: 1 × 10^7^ CFU/mL Pasteurized post-fermentation; lab-scale (1.6 L); triplicate	Fruity, citrus, sour/acidic; honey flavor significantly reduced vs. yeast alone (4.9 vs. 5.9/10, *p* < 0.05) Ranked most acceptable of all 6 NABs (7.0/10) Sourness did not negatively affect panelist acceptance Increased sourness masks the wort-like characteristics of NAB	0.42% 3.54 pH 3.65 TTA (mL 0.1 M NaOH)	Maltotriose not fermented → high residual sugars; 24 h co-fermentation achieved lactic acid comparable to 7-day single-yeast fermentation Diacetyl reduced below threshold (previously above at 0.14 mg/L with yeast alone) Potential route to NAB sour beer LAB did not inhibit yeast kinetics (acid-tolerant strain)	Nyhan et al. (2023)
*Cyberlindnera subsufficiens* C6.1 *+ Lactiplantibacillus plantarum* FST 1.7	Co-fermentation. Inherently very low ethanol producer (maltose- and maltotriose-negative) + LAB acidification. Key metabolites: lactic acid 2.94 g/L (sour beer range); ethyl acetate 19.42 mg/L (doubled vs. C6.1 alone at 9.98 mg/L). Acetic acid depleted >57% by LAB	25 °C, 96 h; 6°P Pilsner wort. Yeast: 1 × 10^6^ CFU/mL; LAB: 1 × 10^7^ CFU/mL. Pasteurized post-fermentation; lab-scale (1.6 L); triplicate	Fruity (pear, melon, pineapple), intense sourness (8.4/10, highest of all conditions, *p* < 0.01); least sweet (*p* < 0.05). Some panelists rated as “too sour”; overall acceptability 6.0/10. Wort-like off-flavors effectively masked	0.32% 3.22 pH 3.77 TTA (mL 0.1 M NaOH)	96 h fermentation required (slow C6.1 growth); pH 3.22 classifies product as sour beer (<3.9). Excess sourness limits acceptance; shorter co-fermentation time or modified wort composition recommended. High residual sugars (maltotriose) do not proportionally increase perceived sweetness. Promising strategy for NAB sour beer production.	Nyhan et al. (2023)
Sugary kefir grain consortium (*Lacticaseibacillus* spp.*, Lentilactobacillus kefiri, Lactococcus lactis, S. cerevisiae, K. lactis +* others)	Post-fermentative enrichment	Kefir grains inoculated into finished commercial NAB; 25 °C; 24 h; aerobic static; 10% (w/v) kefir grain inoculation; 3 commercial Brazilian NABs; residual sugars (~5 °Brix) consumed as substrate; Erlenmeyer flask; lab-scale; triplicate; kefir grains recovered by sieve post-enrichment; stored at 4 °C	No significant sensory differences vs. control across all attributes (appearance, texture, color, flavor, overall acceptability); scores 6.9–8.4 on 9-point hedonic scale (“Like Moderately” to “Like Extremely”); beer color unchanged (EBC/SRM stable at T0 vs. T24)	≤0.5% (maintained non-alcoholic classification); trace ethanol produced during enrichment (0.02–0.03 g/L, ND in controls); original commercial NABs had ND ethanol, pH: ~3.74 → ~3.52, TTA:2.33–2.35% m/v	Post-fermentative approach applied to finished commercial NABs — not a primary fermentation strategy; probiotic viability maintained ≥30 days at 4 °C and 25 °C (LAB ≥10^7^ CFU/mL, yeasts ≥10^4^ CFU/mL; Brazilian MAPA compliant); no VOC/aroma profiling conducted; semi-trained sensory panel (*n* = 100); Brazilian commercial matrix only (sugar/acidity variation not controlled); no *in vivo* health validation; regulatory framework for probiotic labeling on beer not yet established organic acid accumulation enhances antioxidant bioavailability and microbial stability	do Nascimento et al. (2025)

*TTA values are not directly comparable across studies: Nyhan et al. (2023) report mL 0.1 M NaOH per 10 mL sample (endpoint pH 7.0); do Nascimento et al. (2025) report % m/v lactic acid equivalent (phenolphthalein endpoint, ~pH 8.2). Conversion factor: % m/v ≈ mL × 0.09008 (10 mL sample, lactic acid MW = 90.08 g/mol).

The studies summarized in Table 3 indicate that LAB/probiotic-oriented strategies can contribute to NAB quality primarily through controlled acidification and sensory rebalancing. In the co-fermentation experiments of Nyhan et al. (2023), the combination of *Lactiplantibacillus plantarum* with non-*Saccharomyces* yeasts lowered pH, increased lactic acid formation, and reduced the perception of wort-like sweetness. Depending on the yeast partner, this approach either reduced undesirable metabolites such as diacetyl (*Lachancea fermentati*) or enhanced ester-driven fruitiness while masking wort-like character (*Cyberlindnera subsufficiens*). These results suggest that LAB-supported fermentations can shift NAB sensory balance away from the typical sweet–worty profile toward a more acid-balanced and flavor-active product.

A conceptually different strategy was proposed by do Nascimento et al. (2025), who inoculated sugary kefir grains into finished commercial NAB rather than applying LAB during primary wort fermentation. In this case, the process functioned primarily as a post-fermentative enrichment step: pH decreased and organic acids increased while the non-alcoholic classification was maintained and sensory acceptance remained high. This approach therefore represents a functional or probiotic-oriented extension of NAB rather than a fermentation strategy aimed at controlling ethanol formation.

The probiotic-oriented yeast study of Vaštík et al. (2025) was not included in Table 3 because the investigated NAB biofermentative microorganisms (*Pichia manshurica*, *Kluyveromyces lactis*, and *K. marxianus*) function primarily as fermentation yeasts rather than LAB/probiotic-oriented acidification agents. Moreover, the experimental design involved post-fermentation cell inactivation by pascalisation, which moves the concept closer to a functional or postbiotic beverage approach. For this reason, these microorganisms were considered more consistent with the yeast-centered strategies discussed earlier in Supplementary Table S1.

Taken together, LAB/probiotic-oriented approaches expand the technological scope of NAB production beyond simple ethanol limitation. While LAB co-fermentations can improve flavor balance through acidification and masking of wort-like off-flavors, probiotic or kefir-based systems represent a separate functional development pathway. Both directions illustrate how microbial diversity is increasingly being explored to address the sensory and technological limitations of biologically produced non-alcoholic beer.

### 2.3 Process-limited fermentation and integrated production strategies

A review of the literature indicates that, in addition to microbial selection, process-limiting fermentation parameters play a critical role in achieving low or non-alcoholic targets in beer production (Bellut and Arendt, 2019; Nikulin et al., 2022; Jackowski et al., 2023). Within this framework, two main approaches have been proposed: cold contact fermentation and arrested fermentation. Cold-contact fermentation relies on fermentation temperatures near 0 °C to restrict yeast metabolic activity and limit ethanol formation. In a study evaluating four unconventional yeasts isolated from sourdough (*Kazachstania servazzii, Kluyveromyces marxianus, Pichia fermentans* and *Torulaspora delbrueckii*), fermentation at 1.0 ± 0.5 °C demonstrated that *T. delbrueckii* showed the most promising performance, simultaneously limiting ethanol production while reducing wort-derived aldehyde off-flavors (Nikulin et al., 2022). At the 10 L scale, beers produced using *T. delbrueckii* through cold contact fermentation showed no major sensory differences compared to beers produced with a reference lager yeast, despite differences in alcohol content (0.07% vs. 0.28% ABV) (Nikulin et al., 2022).

In contrast, some strains still require arrested fermentation strategies to achieve non-alcoholic targets. In a comparative study of commercial *Saccharomycodes ludwigii* and *Torulaspora delbrueckii* strains, ethanol concentrations of approximately 2.50–2.58% v/v were observed under standard fermentation conditions. Although this represented a 12–15% reduction compared to *Saccharomyces cerevisiae*, these levels remained too high for NAB production, indicating that fermentation must be interrupted at an early stage to meet NAB requirements (Jackowski et al., 2023). These findings demonstrate that the same microbial species can lead to markedly different alcohol outcomes depending on the fermentation strategy applied (Nikulin et al., 2022; Jackowski et al., 2023).

Comparative studies of commercial Pilsner-style NABs further suggest that integrated production strategies may provide more balanced sensory profiles. Beers produced exclusively via restricted fermentation were described as more “worty,” sweet, and full-bodied, whereas beers obtained through physical dealcoholization exhibited thinner and less intense flavor profiles. In contrast, the use of maltose-intolerant yeasts and combined production approaches, such as blending strategies, were associated with more harmonious and multifaceted sensory profiles (Rettberg et al., 2022).

Among the key contributors to off-flavors in NAB are Strecker aldehydes, including 3-methylbutanal, methional, and phenylacetaldehyde. Strategies to reduce their formation include low-temperature fermentation and adsorptive removal techniques. For example, zeolite-based molecular sieves have been reported to reduce Strecker aldehyde concentrations by approximately 43–70% without significantly affecting beer color, bitterness, or pH (Gernat et al., 2020). However, the scalability of such adsorptive approaches and their long-term sensory implications remain insufficiently explored.

Finally, from an industrial perspective, filtration performance, colloidal stability, and shelf-life robustness must also be considered alongside microbial selection. Yeast strain characteristics strongly influence filtration behavior; highly flocculent strains generally produce lower turbidity after filtration, whereas less flocculent yeasts may increase filtration load and require more intensive clarification strategies (Schubert et al., 2025). Moreover, all tested NAB samples showed a tendency toward haze development during storage, highlighting the need for stabilization approaches. In addition to filtration-related challenges, storage conditions also significantly influence the chemical and sensory stability of non-alcoholic beer. Studies have shown that elevated storage temperatures accelerate the formation of aldehydes such as methional, resulting in sensory shifts toward oxidized and wort-like flavor notes (Schubert and Rettberg, 2025). These findings indicate that packaging, storage temperature, and shelf-life management are critical parameters in maintaining the overall quality of NAB products.

## 3 Conclusion

Overall, the literature indicates that the production of NAB through biological approaches relies on the careful integration of microbial selection and process design. Across studies, the ability to limit ethanol formation is most consistently associated with restricted utilization of maltose and maltotriose, low-temperature or process-limited fermentations, and the use of unconventional microorganisms. However, the available evidence also demonstrates that fermentation outcomes cannot be generalized at the species level. Instead, strain-specific physiology, wort composition, fermentation temperature, and inoculation strategy strongly influence both ethanol formation and aroma development. A recurring sensory challenge in biologically produced NAB remains the persistence of sweet or wort-like flavor notes. Several studies suggest that microbial strategies such as LAB-assisted co-fermentation, targeted ester formation, or aldehyde reduction can partially mitigate this limitation, although these approaches may simultaneously shift the sensory profile toward more acidic or unconventional beer styles. As a result, no single microbial group or fermentation strategy consistently resolves the dual objective of ethanol suppression and balanced aroma formation. Beyond fermentation performance, industrial applicability requires attention to downstream processing and product stability. Yeast flocculation behavior influences filtration efficiency and colloidal stability, while storage conditions can accelerate aldehyde accumulation and sensory shifts toward oxidized or wort-like notes. These observations highlight that microbial strategy, process parameters, and post-fermentation handling must be considered as an integrated system. In addition, the increasing exploration of unconventional microorganisms also raises questions related to microbial safety, regulatory status, and consumer acceptance. The use of untraditional yeast and bacterial taxa requires careful evaluation regarding food safety, taxonomic identification, and potential pathogenicity before broader industrial application. Recent studies further indicate that genetically engineered yeast strains can provide promising solutions for more precise control of sugar metabolism and ethanol formation. Nevertheless, their application in brewing remains constrained by regulatory frameworks, regional market restrictions, and persistent consumer skepticism toward genetically modified microorganisms. Consequently, the future of biologically produced NAB is likely to depend not on single-strain solutions, but on integrated fermentation ecosystems that combine strain physiology, process design, and downstream stabilization strategies.

## Supplementary material

SUPPLEMENTARY TABLE S1 Non-Saccharomyces yeast strategies experimentally applied for the biological production of non-alcoholic beer.
