# Supplementary material for: Application of unconventional microorganisms for the production of non-alcoholic beer
Source: Front Microbiol. 2026 May 22;17:1830878. doi: 10.3389/fmicb.2026.1830878 (PMC13236638; doi:10.3389/fmicb.2026.1830878)
Supplement: SUPPLEMENTARY TABLE S1 — Non-Saccharomyces yeast strategies experimentally applied for the biological production of non-alcoholic beer. [file Table_1.docx]

| **Microorganism** | **Mechanism / key metabolites** | **Fermentation conditions** | **Sensory effects** | **ABV (% v/v)** | **Notes / limitations** | **Reference** |
| --- | --- | --- | --- | --- | --- | --- |
| *Hanseniaspora valbyensis* KBI 22.1 | Maltose/maltotriose/sucrose negative; isoamyl alcohol (16.5 mg/L), diacetyl (0.21 mg/L) | 6.6 °P wort; 25 °C; fermentation ceased at 24 h, 8×10⁶ CFU/mL, 2 L lab scale | Wort-like, cereal-like; diacetyl detected by 50% of panel-highest body | 0.35% | Non-flocculent (11%); diacetyl above flavor threshold (>0.1 mg/L); requires maturation step | Bellut et al. (2018) |
| *Hanseniaspora vineae KBI 7*.1 | Maltose/maltotriose/sucrose negative; ethyl acetate (6.00 mg/L — highest among all strains) | 6.6 °P wort; 25 °C; fermentation ceased at 24 h, 8×10⁶ CFU/mL, 2 L lab scale | Black tea, caramel notes; elevated ester not detected sensorially | 0.34% (lowest) | Highest ester producer; sensory panel could not discriminate from commercial AFB strain | Bellut et al. (2018) |
| *Torulaspora delbrueckii* KBI 22.2 | Maltose/maltotriose negative; sucrose positive; acetaldehyde (9.1 mg/L — highest among AFB) | 6.6 °P wort; Mashing 78°C, Hop IBU 10.4 25 °C; fermentation ceased at 48 h, 8×10⁶ CFU/mL, 2 L lab scale | Wort-like, bread-like, honey-like; lowest diacetyl | 0.50% | Non-flocculent (17%); highest acetaldehyde among AFB strains | Bellut et al. (2018) |
| *Torulaspora delbrueckii (White Laboratories)* | Maltose/maltotriose-negative under tested conditions; glucose/fructose/sucrose only; fruity-floral ester profile reported in other studies; Ethyl caproate: ~0; Isoamyl alcohol **1.17–1.60 mg/L** (the highest group); 2-methylbutan-1-ol ~0.15–0.23 mg/L | 4.5°P and 9°P wort; 10 BU; 20°C; 108 h; 1×10⁷ cells/mL; pilot scale (20-gallon); thermal pasteurization (400 PU); triplicate sensory | Raisin/dried fruit (the highest score) —Fruity character; NAB-compliant at 4.5°P only; aroma profile not separately reported per wort strength | 4.5°P: 0.41%; 9°P: >0.5% (NAB limit exceeded) | NAB-compliant only at 4.5°P; note that *T. delbrueckii* is typically maltose-positive — behavior here may reflect strain-specific variation or process conditions | Maust et al. (2025) |
| *Zygosaccharomyces bailii* KBI 25.2 | Maltose/maltotriose negative; sucrose positive; balanced volatile profile | 6.6 °P wort; 25 °C; fermentation ceased at 72 h, 8 × 10⁶ CFU/mL, 2 L lab scale | Slightly grassy, fruity, white wine notes; lowest wort-like score among AFB | 0.42% | Slowest fermentation (72 h); identified as most promising sensory candidate among non-*Saccharomyces* strains | Bellut et al. (2018) |
| *Zygosaccharomyces kombuchaensis* KBI 5.4 | Maltose/maltotriose negative; sucrose positive; diacetyl (0.15 mg/L) | 6.6 °P wort; 25 °C; fermentation ceased at 72 h, 8 × 10⁶ CFU/mL, 2 L lab scale | Wort-like; diacetyl flavor detected by 50% of panel | 0.48% | Diacetyl above flavor threshold; slowest metabolism along with KBI 25.2 | Bellut et al. (2018) |
| *Saccharomycodes ludwigii* TUM SL 17 | Maltose/maltotriose negative; sucrose positive; balanced secondary metabolite profile | 6.6 °P wort; 25 °C; fermentation ceased at 48 h, 8×10⁶ CFU/mL, 2 L lab scale | Wort-like, bread-like, honey-like (90% of panel) | 0.50% | reference NAB yeast | Bellut et al. (2018) |
| *Saccharomycodes ludwigii* TUM SL 17 | Maltose/maltotriose negative; 2-phenylethanol (6.6 mg/L); low ester profile | 7.0 °P unhopped wort; 20 °C; 144 h; 15×10⁶ cells/mL; 2 L lab scale | Honey, apple, caramel-like; non-significantly fruity (8/10 panel) | 0.50% | DLG score 4.16 (lower end of range); least fruity among tested strains; reference strain for comparison | Methner et al. (2022) |
| *Saccharomycodes ludwigii* (WhiteLaboratories)-NA all day | Maltose/maltotriose-negative; glucose/fructose/sucrose only; characteristically clean fermentation profile with low ester production; quantitative; very low ethyl caproate; Isoamyl alcohol 0.81–1.31 mg/L; 2-phenylethanol ~0.22–0.28 mg/L | 4.5°P and 9°P wort; 10 BU; 20°C; 108 h; 1×10⁷ cells/mL; pilot scale (20-gallon); thermal pasteurization (400 PU); triplicate sensory | low overall aroma + malt/cereal character; NAB-compliant at 4.5°P only; sensory differentiation between wort strengths not separately reported; dried fruit /cereal/ wort | 4.5°P: 0.28%; 9°P: 0.73% (NAB limit exceeded) | NAB-compliant only at 4.5°P; wort strength critical for this strain | Maust et al. (2025) |
| *Cyberlindnera subsufficiens* C6.1 | Maltose/maltotriose-negative; high isoamyl acetate (0.80 mg/L); ethyl acetate (12.0 mg/L); glucophilic — residual fructose | 7.0 °P wort; 17 °C; 10⁷ cells/mL; 13 days; pilot scale (60 L) | Fruity (pear, banana, mango, maracuja); significantly reduced wort-like aroma vs. commercial NABs (p ≤ 0.001) | 0.36% | Low flocculation (32%); optimized via RSM; fruity aroma masks wort-like off-flavor; pasteurization required (23 PU); unidentified compounds likely contribute to fruitiness | Bellut et al. (2019) |
| *Cyberlindnera sufficiens* C6.1 | Maltose/maltotriose-negative; ethyl acetate 9.98 mg/L; acetic acid present; pH 4.60 | 6°P unhopped wort, 25°C, 96 h; 1×10⁶ CFU/mL; lab-scale (1.6 L); triplicate; pasteurised post-fermentation | Fruity wort-like off-flavors partially retained | 0.26% | lowest ABV of all conditions tested; slow growth requires 96 h; partially retained when fermented alone | Nyhan et al. (2023) |
| *Cyberlindnera saturnus* (strains TUM 247, CBS 4549, CSa1) | Maltose/maltotriose negative; high isoamyl acetate (3.1–7.5 mg/L; up to 6.25× threshold); 2-phenylethyl acetate (0.35–1.30 mg/L) | 7.0 °P unhopped wort (malt extract); 20 °C; 144 h; 15×10⁶ cells/mL; 2 L lab scale | Cool mint sweets (50–80% of panel), pear, banana; red berry (strain 247 only, 50%); solvent-like note detected; significantly fruity (10/10 panel, α=0.05) | 0.28–0.46% | Highest DLG scores among all tested strains (4.54 for TUM 247 and CBS 4549); isoamyl acetate above flavor threshold — may cause solvent-like off-note at high concentrations; unhopped wort; pasteurization recommended due to residual sugars | Methner et al. (2022) |
| *Kluyveromyces marxianus* 653 | Maltose-negative; diacetyl above threshold (0.26 mg/L); 2-phenylethyl acetate (0.35–1.30 mg/L) | 7.0 °P unhopped wort; 20 °C; 144 h; 15×10⁶ cells/mL; 2 L lab scale | Red berry, honey, stone fruit, apple; slightly buttery (diacetyl); significantly fruity (9/10 panel) | 0.37% | DLG score 4.38; diacetyl above threshold (0.26 mg/L) — maturation step may be required; broad fruity profile noteworthy | Methner et al. (2022) |
| *Kluyveromyces marxianus* CCY 029-008-010 | Maltose-negative, lactose-positive; Crabtree-negative, thermotolerant (up to 45°C); strong β-glucosidase activity; POF+; ethyl acetate (374 µg/L), 2-phenylethyl acetate (167 µg/L), 2-phenylethanol (1198 µg/L), 4-vinylguaiacol (681 µg/L); no acetic acid; diacetyl not detected | 8°P hopped wort; 20°C, 2 days + 3°C, 3 weeks maturation; 1×10⁶ cells/mL; 480 mL PET flasks; triplicate; pascalised (400 MPa, 3 min) | No formal sensory panel conducted; VOC profile suggests fruity character with clove note (4-VG); higher ethyl acetate vs. *K. lactis*; diacetyl absent | 0.14% | Thermotolerance (up to 45°C) not exploited under tested conditions; minimal glucose consumption under closed fermentation (Kluyver effect); pH above typical beer range (5.36); pascalisation inactivates cells; lab-scale only | Vaštík et al. (2025) |
| *Saccharomycopsis fibuligera* Lu27 | Weak maltose utilization via extracellular glucoamylase (~10%); diacetyl above threshold (0.21 mg/L); broad ester profile | 7.0 °P unhopped wort; 20 °C; 144 h; 15×10⁶ cells/mL; 2 L lab scale | Red berry, stone fruit (plum), honey, apple; significantly fruity (9/10 panel) | 0.47% | DLG score 4.32; not strictly maltose-negative (glucoamylase activity); diacetyl above threshold; unusual morphology in selective media (gelatinous yeast balls) complicates analysis | Methner et al. (2022) |
| *Starmerella bombicola CCY 029-180-002* | Maltose/maltotriose-negative; high glycerol (1.0 g/L in wort); acetic acid producer (1.1 g/L); low ester profile | 10 °P hopped wort; 15 °C; 10 days; 10⁶ cells/mL; 45 mL lab scale | Neutral aroma; no notable fruity character; no 3-methylbutyl acetate detected | 0.45% | First reported use of *S. bombicola* in beer fermentation; high glycerol may contribute to beer body; VOC analysis conducted under unified conditions (12 °C, 10 days; ABV: ~0.58% v/v under those conditions); **co-fermentation with *L. jadinii* suggested** to enhance aroma | Vaštík et al. (2022) |
| *Lindera jadinii CCY 029-038-042* | Maltose/maltotriose-negative; 3-methylbutyl acetate (1.6 mg/L — at flavor threshold); ethyl acetate (12.4 mg/L); acetic acid (1.1 g/L) | 10 °P hopped wort; 10 °C; 10 days; 10⁶ cells/mL; 45 mL lab scale | Banana-like flavor; fruity aroma | 0.09% v/v | VOC analysis conducted under unified conditions (12 °C, 10 days; ABV: ~0.51% v/v); banana character attributed to 3-methylbutyl acetate at threshold level; shorter fermentation or higher temperature risks exceeding 0.5% ABV | Vaštík et al. (2022) |
| *Pichia kluyveri CCY 029-009-046* | Maltose-negative; sucrose positive; 3-methylbutyl acetate (2.3 mg/L — above threshold); ethyl acetate (5.4 mg/L); hexanoic acid (3.4 mg/L) | 10 °P hopped wort; wide range of conditions suitable (10–25 °C) (5-15 days); 10⁶ cells/mL; 45 mL lab scale | Banana aroma not prominent despite 3-methylbutyl acetate above threshold; relatively neutral overall | 0.34% (15 °C, 10 days) | VOC analysis conducted under unified conditions (12 °C, 10 days; ABV: ~0.52% v/v); most flexible fermentation conditions among tested strains; disconnect between chemical analysis and sensory perception noted by authors | Vaštík et al. (2022) |
| *Lindnera mrakii* NCYC 500 | Maltose/maltotriose/sucrose-negative (no β-fructosidase); high ethyl acetate (52.18 mg/L — ~2× flavor threshold); low total higher alcohols (11.19 mg/L); DMS 20.30 mg/L (above threshold) | 12°P hopped wort; 14°C; 10–12 days; 1 × 10⁶ cells/mL; 2 L EBC tubes; initial wort pH 4.90; biological duplicates | Worty, malty, cereal-like; glue-like off-flavor (elevated ethyl acetate); cooked vegetable note (elevated DMS); pronounced sweetness due to residual maltose | 0.30% | Ethyl acetate above flavor threshold (52.18 vs. 25–30 mg/L); DMS above threshold (20.30 mg/L); authors conclude **unsuitable as single-fermentation yeast**; co-culture approach suggested | Simões et al. (2023) |
| *Pichia kluyveri* NEER (Chr. Hansen SmartBev™) | Maltose/maltotriose/sucrose-negative (no β-fructosidase); high isoamyl acetate (9.06 mg/L — well above 1.2–2.0 mg/L threshold); ethyl acetate (32.59 mg/L — above threshold); lowest attenuation among all strains (RDF 2.18%) | 12°P hopped wort; 14°C; 10–12 days; 1 × 10⁶ cells/mL; 2 L EBC tubes; initial wort pH 4.90; biological duplicates | Worty, malty, cereal-like; banana character chemically expected (isoamyl acetate) but not prominently perceived sensorially; pronounced sweetness; sensory-chemical disconnect noted by authors | 0.17% (lowest among all tested strains) | Commercial strain (Chr. Hansen SmartBev™); distinct from P. kluyveri CCY 029-009-046 Vaštík et al. (2022); isoamyl acetate and ethyl acetate both above flavor threshold; sucrose not fermented; sensory-chemical disconnect observed | Simões et al. (2023) |
| *SMARTBEV™ NEER® (Pichia kluyveri; Chr. Hansen)* | maltose-negative/P. Glucose/fructose fermentation only; high acetate ester production: ethyl acetate 2312 µg/L, isoamyl acetate 1398 µg/L, phenylethyl acetate 603 µg/L, isobutyl acetate 14.8 µg/L; sum esters 4337 µg/L; sum higher alcohols 8.7 ± 1.0 mg/L (low); isobutanol dominant higher alcohol (3.9 mg/L); diacetyl 20.5 ± 18.0 µg/L (above 17 µg/L threshold, masked by high esters); aldehyde reduction 92.7% (sum aldehydes 20.6 ± 2.0 µg/L); glycerol 205 ± 7 mg/L; FAN assimilation −1 ± 5% (minimal/negligible); tannic acid (Brewtan®B) added during mashing to minimize sulphury off-flavors | ~20 EBU, 6.22 ± 0.16 °P; mash 72°C/60 min; wort pH pre-adjusted to 4.5 with lactic acid; 20°C; 6 days fermentation + 7 days maturation at 3°C; 2×10⁵ CFU/mL; periodic agitation (every 2–8 h) to keep yeast in suspension; 20 L conical stainless-steel fermenter; filtration (2 µm), CO₂ saturation (5.6 g/L), batch pasteurization (50 PU); duplicate fermentations | Fruity significantly above panel mean (RATA 1.91 — highest among all tested yeasts); floral above mean (0.86); solvent-like above mean (0.45, isoamyl acetate-driven); sweet above mean (1.52); worty present but below average (0.95); bitter and astringency below mean; watery not prominent (high residual extract) | 0.16% | Sucrose not fermented — residual sucrose 1.86 ± 0.79 g/L contributes to sweetness; diacetyl above sensory threshold but sensorially masked by high acetate esters; solvent-like character may limit beer-style acceptability; tannic acid addition required for sulphury off-flavor control (strain-specific requirement not needed for Poly/Punch); low glycerol limits body; untrained industrial panel (n=44); RATA methodology; 20 L pilot scale  polyphenols 152 ± 3 mg/L (elevated due to Brewtan®B addition) | Myncke et al. (2025) |
| *Pichia kluyveri* (Novonesis) -NEER Punch | Maltose/maltotriose-negative; glucose/fructose/sucrose only; Ethyl caproate 4.5°P: **~0** → 9°P: 68.5 µg/L; Isoamyl alcohol ~0.74–0.85 mg/L | 4.5°P and 9°P wort; 10 BU; 20°C; 108 h; 1×10⁷ cells/mL; pilot scale (20-gallon); thermal pasteurization (400 PU); triplicate sensory | Generally characterized as a low aroma sample. Low overall aroma with slight grape character. NAB-compliant at both wort strengths. Aroma intensity increases with wort strength | 4.5°P: 0.12%; 9°P: 0.32% — NAB-compliant at both wort strengths | Limited ester expression under the tested conditions, likely due to lack of aeration/agitation during fermentation (manufacturer-recommended aeration/agitation was not applied in this study). | Maust et al. (2025) |
| SMARTBEV™ NEER*®* Punch *(Pichia kluyveri;* Chr. Hansen) | maltose-negative; Glucose/fructose fermentation only; sucrose not fermented; residual maltose 19.24 ± 2.51 g/L; acetate ester production: ethyl acetate 2714 µg/L, isoamyl acetate 989 µg/L, phenylethyl acetate 1185 µg/L (highest phenylethyl acetate among *P. kluyveri* strains); isobutyl acetate 31.7 µg/L; sum esters 4938 µg/L; sum higher alcohols 9.0 ± 1.2 mg/L; diacetyl 53.1 ± 21.8 µg/L (highest among all tested yeasts); aldehyde reduction 94.6%; glycerol 202 ± 7 mg/L; FAN assimilation 27 ± 23% | ~20 EBU,  6.56 ± 0.25 °P; mash 72°C/60 min; wort pH pre-adjusted to 4.5 with lactic acid; 20°C; 6 days fermentation + 7 days maturation at 3°C; 2×10⁵ CFU/mL; periodic agitation (every 2–8 h) — continuous agitation in one replicate caused ABV increase (0.40 vs. 0.11% v/v) demonstrating agitation sensitivity; 20 L conical stainless-steel fermenter; filtration (2 µm), CO₂ saturation (5.6 g/L), batch pasteurization (50 PU); duplicate fermentations | Fruity above mean (RATA 1.25); floral above mean (0.86, tied with NEER — highest phenylethyl acetate drives floral character); sweet above mean (1.41); worty below mean (0.84 — lowest worty score among all tested yeasts); bitter and astringency below mean; cooked vegetables not elevated (unlike Poly) | 0.26%  (high SD due to agitation-sensitive replicate: 0.11 vs. 0.40%) | ABV highly sensitive to agitation intensity, continuous vs. periodic mixing caused ~4× increase in one replicate; highest diacetyl among all yeasts (53.1 µg/L) but sensorially masked by esters; sucrose residual 1.94 ± 1.38 g/L; tannic acid not required; agitation protocol critical for reproducibility; low glycerol reduces body; untrained industrial panel (n=44); RATA methodology  **acetate-ester dominant maltose-negative yeasts** | Myncke et al. (2025) |
| SMARTBEV™ NEER® Poly *(Pichia kluyveri;* Chr. Hansen) | maltose-negative; Glucose/fructose fermentation only; residual maltose 19.09 ± 0.91 g/L; high acetate ester production: ethyl acetate 3243 µg/L, isoamyl acetate 1454 µg/L, phenylethyl acetate 937 µg/L, isobutyl acetate 62.4 µg/L (highest among P. kluyveri strains); sum esters 5707 µg/L (highest in study); sum higher alcohols 8.7 ± 3.1 mg/L; diacetyl 50.1 ± 54.0 µg/L (above threshold, high variability between replicates); aldehyde reduction 94.2%; glycerol 136 ± 85 mg/L (lowest among all tested yeasts); FAN assimilation 0 ± 1% | ~20 EBU, 6.43 ± 0.10 °P; mash 72°C/60 min; wort pH pre-adjusted to 4.5 with lactic acid; 20°C; 6 days fermentation + 7 days maturation at 3°C; 2×10⁵ CFU/mL; periodic agitation (every 2–8 h); 20 L conical stainless-steel fermenter; filtration (2 µm), CO₂ saturation (5.6 g/L), batch pasteurization (50 PU); duplicate fermentations; tannic acid NOT added (unlike NEER) | Fruity above mean (RATA 0.80); floral comparable to NEER (0.36); cooked vegetables significantly above panel mean (1.34 — highest in study, sulfuric compound suspected, not DMS-driven); sweet above mean (1.50); worty intermediate (1.36); sensory differentiation from NEER and Punch despite similar VOC profile — cooked vegetable character distinguishes Poly | 0.12% | Highest sum esters and lowest glycerol in study; cooked vegetable off-flavor not explained by DMS, sulfuric compound(s) unidentified; high diacetyl variability between replicates (50.1 ± 54.0 µg/L) indicates fermentation inconsistency; sucrose residual 2.69 ± 0.26 g/L; tannic acid omitted, confirms this additive not required for Poly; low glycerol reduces body; continuous vs. periodic agitation shown to affect ABV and glycerol; untrained industrial panel (n=44); RATA methodologypH 4.62 | Myncke et al. (2025) |
| *Lachancea fermentati* KBI 12.1 | Maltotriose-negative; 2-phenylethanol 3.50 mg/L; diacetyl 0.14 mg/L (above 0.1 mg/L threshold); acetoin 2.69 mg/L | 6°P unhopped wort; 25°C, 24 h; 1×10⁶ CFU/mL; lab-scale (1.6 L); triplicate; pasteurised post-fermentation | Honey flavor prominent (5.9/10); diacetyl above sensory threshold (0.14 mg/L) → buttery off-note; wort-like characteristics partially retained; overall acceptability reduced vs. co-fermentation condition | 0.45% | diacetyl above sensory threshold when fermented alone | Nyhan et al. (2023) |
| *Pichia manshurica* CCY 039-063-001 (PM1)  CCY 039-063-004 (PM2) | Maltose/lactose-negative; glucose-only fermenter; weak/delayed β-glucosidase activity; POF+ (4-vinylguaiacol: 650–628 µg/L); higher alcohols: 2-phenylethanol (1730–2377 µg/L), 3-methyl-1-butanol (615–1196 µg/L), 2-methyl-1-butanol (364–828 µg/L); ethyl acetate (19–24 µg/L — low); no acetic acid, no diacetyl detected | 8°P hopped wort, 20°C, 2 days + 3°C, 3 weeks maturation; 1×10⁶ cells/mL; 480 mL PET flasks; triplicate; pascalised post-fermentation (400 MPa, 3 min) | No formal sensory panel conducted; VOC profile indicates clove-like aroma (4-VG above threshold) **suited to wheat-style NAB**; higher alcohols below flavor thresholds; diacetyl absent | PM1: 0.04%; PM2: 0.07% | First reported use of *P. manshurica* in beer fermentation; minimal glucose consumption under closed fermentation conditions (Kluyver effect likely); pascalisation inactivates cells → postbiotic potential; viability at 37°C >97% (GI tract simulation); pH 3 viability low (16–17%) — limits probiotic transit claim; no sensory panel; lab-scale only; wheat-style NAB niche suggested. | Vaštík et al. (2025) |
| *Kluyveromyces lactis* CCY 026-012-002 | Maltose-negative, lactose-positive; strong β-glucosidase activity (hop aglycone release); POF+; 2-phenylethyl acetate (358 µg/L), ethyl acetate (212 µg/L), 2-phenylethanol (1373 µg/L), 4-vinylguaiacol (630 µg/L); no acetic acid; diacetyl not detected | 8°P hopped wort (Pilsen malt, Saaz hops); 20°C, 2 days + 3°C, 3 weeks maturation; 1×10⁶ cells/mL; 480 mL PET flasks; triplicate; pascalised (400 MPa, 3 min) | No formal sensory panel conducted; VOC profile suggests fruity-floral character with clove note (4-VG); diacetyl absent | 0.13% | minimal **glucose** consumption under closed fermentation conditions — *K. lactis* glucose fermentation is oxygen-dependent (Kluyver effect); pH above typical beer range (5.41) due to short fermentation; strong β-glucosidase activity potentially enhances hop-derived aroma complexity; pascalisation inactivates cells; lab-scale only | Vaštík et al. (2025) |
| *Hanseniaspora uvarum* (Escarpment Laboratories)-NAY | Maltose/maltotriose-negative; glucose/fructose/sucrose only; ester and higher alcohol production; aldehyde reduction capacity strain-dependent; Ethyl caproate 4.5°P: **25.4 µg/L**→ 9°P: 9.0 µg/L (fruity, melon driver); isoamyl alcohol ~0.24–0.44 mg/L | 4.5°P and 9°P wort; 10 BU; 20°C; 108 h; 1×10⁷ cells/mL; pilot scale (20-gallon); thermal pasteurization (400 PU); triplicate sensory | Fruity character,banana- melon dominant; NAB-compliant at both wort strengths; wort-like off-flavor intensity not separately reported | 4.5°P: 0.03%; 9°P: 0.17% — NAB-compliant at both wort strengths | NAB-compliant at both wort strengths. low attenuation and strong ester production allow fruity NAB profiles while maintaining very low alcohol levels. | Maust et al. (2025) |
